# Effects of neighbourhood-level educational attainment on HIV prevalence among young women in Zambia

**DOI:** 10.1186/1471-2458-9-310

**Published:** 2009-08-25

**Authors:** Nkomba Kayeyi, Ingvild F Sandøy, Knut Fylkesnes

**Affiliations:** 1 Centre for International Health, University of Bergen, Overlege Danielssens hus, 5th floor, Årstadveien 21, N-5009 Bergen, Norway

## Abstract

**Background:**

Investigations of the association between socio-economic position indicators and HIV in East, Central and Southern Africa have chiefly focused on factors that pertain to individual-level characteristics. This study investigated the effect of neighbourhood educational attainment on HIV prevalence among young women in selected urban and rural areas in Zambia.

**Methods:**

This study re-analysed data from a cross-sectional population survey conducted in Zambia in 2003. The analyses were restricted to women aged 15–24 years (n = 1295). Stratified random cluster sampling was used to select 10 urban and 10 rural clusters. A measure for neighbourhood-level educational attainment was constructed by aggregating individual-level years-in-school. Multi-level mixed effects regression models were run to examine the neighbourhood-level educational effect on HIV prevalence after adjusting for individual-level underlying variables (education, currently a student, marital status) and selected proximate determinants (ever given birth, sexual activity, lifetime sexual partners).

**Results:**

HIV prevalence among young women aged 15–24 years was 12.5% in the urban and 6.8% in the rural clusters. Neighbourhood educational attainment was found to be a strong determinant of HIV infection in both urban and rural population, i.e. HIV prevalence decreased substantially by increasing level of neighbourhood education. The likelihood of infection in low vs. high educational attainment of neighbourhoods was 3.4 times among rural women and 1.8 times higher among the urban women after adjusting for age and other individual-level underlying variables, including education. However, the association was not significant for urban young women after this adjustment. After adjusting for level of education in the neighbourhood, the effect of the individual-level education differed by residence, i.e. a strong protective effect among urban women whereas tending to be a risk factor among rural women.

**Conclusion:**

The findings suggested structural effects on HIV prevalence. Future research should include more detailed mapping of neighbourhood factors of relevance to HIV transmission as part of the effort to better understand the causal mechanisms involved.

## Background

Investigations of the association between socio-economic position indicators and HIV have chiefly focused on factors that pertain to individual-level characteristics. In a systematic review of HIV studies in East, Central and Southern Africa, Wojcicki et al. (2005) found that out of the 36 studies examined, only two studies [1,2] had taken ecological or neighbourhood variables into consideration [3]. Further evidence points to the fact that studying individual characteristics such as demographic, biologic, personality or behaviour factors alone can only explain a part of the complex set of factors that contribute to poor health [4-6]. Neighbourhoods represent both social networks and physical places through which diseases spread [4], and ignoring them may lead to incomplete understanding of determinants of disease in individuals and in the population [5].

Multi-level studies in public health have focused on a range of different public health outcomes, for instance, related to all-cause mortality, cardiovascular disease, infant and child health, women's health, mental health and health behaviour [7-12]. In the past years, there has been an increased interest in neighbourhood or ecological variables in Africa, such that more studies have started to investigate their effect on HIV prevalence [13,14]. The associations studied have been mainly between neighbourhood variables such as socio-economic position, socio-economic activities and HIV prevalence. These studies have found neighbourhood variables to be associated with HIV prevalence.

Individual-level epidemiological surveys on the association between educational attainment and HIV infection have found different patterns depending on the stage of the epidemic. Studies conducted before 1996 in sub-Saharan Africa found higher educational attainment to be positively associated with HIV infection [15-17]. The explanation for these findings was probably that higher educational achievement was associated with higher wealth, increased mobility and multiple sexual partners [15,18]. Later, serial cross-sectional studies have shown declines in the HIV prevalence among highly educated persons, particularly in those aged 15–24 years [17,19-23]. It is likely that increased information, knowledge and awareness, might have had a positive impact earlier among educated persons than those who were illiterate and poor, in terms of delaying sexual debut, reducing the number of partners, and increasing condom use [24,25].

Conversely, there have been few studies that have investigated the effect of neighbourhood educational attainment or socio-economic position on HIV prevalence. In the present study, HIV survey data from selected urban and rural neighbourhoods in Zambia were re-analysed using multi-level modelling techniques to investigate the relationship between HIV prevalence and educational attainment at both the neighbourhood and individual levels.

## Methods

### Population and sampling procedures

The investigation is based on data collected in a population-based cross-sectional survey undertaken in Zambia in 2003. Stratified random cluster sampling was used to select ten clusters in Chelston (urban neighbourhood in Lusaka) and ten in Kapiri Mposhi district (rural neighbourhoods). Each cluster in the study corresponds to a census tract or standard enumeration area (SEA), which is the lowest unit of data collection in a Census of Zambia. It should be noted that the terms cluster and neighbourhood are used interchangeably in this study.

All persons in the selected clusters aged between 15–59 years were invited to participate in the study. A total of 6791 persons were listed and 4751 persons (70%) completed the structured interview and provided saliva for HIV testing. Two call-backs were made in order to catch individuals who were absent during the first visit to the household. Non-participation was attributed to absence of some listed persons during the canvassing (19.7%), interview refusals (3.4%) and refusal to test for HIV infection (6.6%). This paper is restricted to young women aged between 15–24 years (n = 1295). There were 840 young women aged 15–24 who participated and were tested for HIV in Chelston, whereas the corresponding number in Kapiri Mposhi was 456.

### Laboratory Analysis

In order to ascertain the HIV status of the participants, the study collected saliva samples from willing participants. Participants' saliva samples were tested by use of a rapid test kit BIONOR HIV 1 & 2 (BIONOR AS, Skien, Norway). For quality assurance, 10 percent of negative samples and 10 percent positive samples were retested at the national reference laboratory at the University Teaching Hospital in Lusaka.

### Conceptual Framework

The analysis and interpretation of the data was based on the proximate-determinants framework for HIV infection [26]. Key to the framework is the assumption that underlying variables influence the proximate variables, which in turn have a direct effect on the biological mechanisms behind the acquisition of HIV. The underlying variables include socio-economic context of the neighbourhood (neighbourhood educational attainment, neighbourhood wealth index and neighbourhood employment rate are all used as a proxy of this in the current study), individual education, marital status, wealth index, student status (still student) and mobility. In order to affect the biological pathways to HIV infection, the underlying variables must operate through proximate variables, for example, age at sexual debut, being sexually active, and number of lifetime sexual partners. Distinguishing underlying variables from proximate variables is important for the conceptualization of causal pathways leading to HIV infection.

### Variables

The neighbourhood variables were constructed based on the aggregation method in SPSS 15. This is a procedure that allows summarizing of a dataset or a variable by collapsing it into summary statistics (for example, mean, median, sum and standard deviation) on the basis of a break variable [27]. A break variable is a variable for which summary statistics are to be computed, for instance, clusters (SEAs) in this study. Each of the neighbourhood-level measures were grouped into three levels, the lowest level denoting low mean educational level, low wealth level or low employment level (i.e. high unemployment), and high denoting high mean educational level, high wealth level or high employment level. Values in-between were considered "middle".

To measure the variable educational attainment, respondents were asked how many years they had spent in school, and they gave the answer in actual number of years. Different cut-off points had to be used for the urban compared with the rural setting due to the marked difference in the distribution of educational attainment. Zambia's educational system is divided into four levels, that is, primary school (grade 1 – 7), junior secondary school (grade 8 – 9), senior secondary school (grade 10 – 12), and tertiary education (above grade 12). However, we set the following cut-off points for individual-level educational attainment in the Chelston neighbourhoods to avoid making one group very small: 0 – 7 years (low educational level), 8 – 11 years (middle educational level) and 12 years and above (high educational level). Since the reported number of years in school was skewed towards primary education in Kapiri Mposhi, the following groupings were employed: 0 – 4 years (low educational level), 5–7 years (middle educational level) and 8 years and above (high educational level). The neighbourhood-level educational attainment was estimated by calculating the mean number of years in school for all respondents in the neighbourhood (aged 15 – 59 years). The neighbourhoods were then grouped into three socio-economic classes: low, middle and high. This was done separately for the urban and rural neighbourhoods. Using the proportion with secondary school or higher level of education gave the same categorization of clusters as when employing mean years-in-school.

The variable employment was based on a question on current employment status, and the response options were 'unemployed', 'unpaid family worker', 'self-employed', 'employee', and 'employer'. After assessing the data, we reduced the scale from five items to two items. 'Unemployed' and 'unpaid family worker' were combined and the new category was called 'unemployed', and 'self-employed', 'employee' and 'employer' were merged to form a category called 'employed'. To be considered employed, respondents were supposed to have a job that paid them money or to be engaged in some form of business that earned them an income. Otherwise, they were considered unemployed. Thus housewives were considered to be unemployed. Students were not included in the two categories. For each cluster the proportion of unemployed respondents aged 15–59 was calculated, and the clusters were grouped into three categories; low, medium and high employment rate (i.e. the inverse of unemployment in order to obtain a "high"-category that corresponded to higher socioeconomic status). The categorization was done separately for the urban and rural areas, and cut-off points were selected that resulted in groups of similar sizes.

The individual level wealth index was derived using principle component analysis based on six household items, i.e. electricity, refrigerator, radio, bicycle, plough and donkey. We created two separate wealth indices for the rural and the urban areas to account for the fact that different items were of different importance for urban and rural residents. Three individual wealth index categories were made based on the values of Component 1 obtained in the principal component analyses. In addition, the values of Component 1 for all respondents aged 15–59 were used to calculate the mean wealth status of each cluster using the aggregation procedure, and three categories representing the relative wealth status of the neighbourhoods were then created (this was done separately for the urban and rural clusters).

The variable travel was measured by a question on trips made in the past year that involved absence from home for several days. The response options were 'never', 'sometimes', 'often', and 'very often', and the variable was dichotomised into 'never/seldom' and 'often' (often and very often).

### Statistical analysis

All analyses were restricted to young women aged between 15–24 years with known HIV status. Young women were selected based on the premise that they are highly vulnerable to HIV infection as indicated by the very high HIV prevalence relative to young men [23]. Furthermore, the bulk of infections in this group are relatively recent and mortality is low. The analyses controlled for the effect of the potential confounder age, which was adjusted for as a linear effect.

All analyses were conducted using Stata version 10.1 (College Station, Texas, USA), except for the aggregation procedures and the linear-by-linear test for which we used SPSS 15. The distribution and age-adjusted associations (based on multilevel mixed-effect logistic regression) of underlying and proximate variables to HIV prevalence are shown in Table 1. Multilevel mixed-effect logistic regression estimates both fixed and random effects and takes account of the clustering of data. Variables that were significantly associated (p < 0.05) with HIV in the bivariate analysis in either the urban or the rural areas were included in the multivariate analyses (in Tables 2 and 3). In addition, Pearson's correlation test was conducted to determine the correlation between cluster-level educational attainment, cluster-level wealth index, cluster employment rate, presence of electricity in the cluster, and individual educational attainment. The results indicated that cluster-level wealth; cluster employment and electricity were highly correlated with cluster educational level in the rural neighbourhoods. To avoid multi-collinearity during modelling, cluster educational attainment was the only cluster-related variable retained in the final multivariate model.

**Table 1:** Bivariate and age-adjusted distribution of underlying and proximate factors of HIV infection in young women by urban and rural residence

Variable	**Urban**						**Rural**					
	
	All women			Sexually active women			All women			Sexually active women		
	No.	HIV %	AOR	No.	HIV %	AOR	No.	HIV %	AOR	No.	HIV %	AOR
Cluster education												
Low	397	15.9	1.00	245	22	1.00	159	10.7	1.00	131	13.0	1.00
Middle	195	12.3	0.77 (0.46–1.29)	96	21.9	1.02 (0.57–1.80)	136	5.9	0.51 (0.21–1.23)	125	6.4	0.47 (0.19–1.14)
High	248	7.3	**0.42 (0.24–0.74)**	127	11.8	**0.47 (0.25–0.88)**	161	3.7	**0.32 (0.12–0.85)**	108	5.6	**0.37 (0.14–0.97)**
Cluster wealth Index												
Low	264	14.4	1.00	171	19.9	1.00	175	10.3	1.00	112	14..3	1.00
Middle	244	11.5	0.86 (0.43–1.74)	128	18.8	0.94 (0.47–1.88)	120	5.8	0.44 (0.19–1.02)	144	6..3	0.44 (0.19–1.03)
High	332	11.7	0.85 (0.43–1.68)	169	18.9	0.90 (0.45–1.78)	161	3.7	0.37 (0.11–1.32)	108	5.6	0.47 (0.13–1.66)
Cluster employment												
Low	268	13.3	1.00	178	18.4	1.00	138	8	1.00	111	9.9	1.00
Middle	244	12.3	0.93 (0.46–1.92)	130	20.8	1.19 (0.59–2.40)	156	7.7	0.91 (0.38–2.17)	136	8.8	0.86 (0.36–2.04)
High	328	11.6	0.85 (0.42–1.74)	168	18.9	1.01 (0.51–2.01)	162	4.9	0.56 (0.21–1.44)	117	8.5	0.61 (0.23–1.58)
Education												
Low	174	20.1	1.00	101	27.7	1.00	151	7.9	1.00	125	9.6	1.00
Middle	313	14.7	0.69 (0.41–1.14)	158	25.3	0.84 (0.47–1.50)	184	6.5	0.82 (0.34–2.01)	155	7.7	0.80 (0.34–1.86)
High	352	6.8	**0.17 (0.10–0.32)**	209	10.5	**0.25 (0.13–0.48)**	121	5.8	0.75 (0.23–2.35)	84	8.3	0.83 (0.31–2.21)
Wealth index												
Low	227	12.3	1.00	153	15.7	1.00	203	8.4	1.00	173	9.8	1.00
Middle	495	12.5	1.13 (0.68–1.88)	255	21.6	1.51 (0.87–2.62)	136	4.4	0.53 (0.20–1.40)	113	5.3	0.52 (0.20–1.38)
High	117	12.8	1.16 (0.58–2.34)	60	18.3	1.21 (0.54–2.71)	116	6.9	0.95 (0.35–2.56)	78	10.3	1.04 (0.40–2.70)
Current student												
Not student	505	16.6	1.00	352	21.9	1.00	370	8.4	**a**	324	9.6	**a**
Student	332	6.3	**0.47 (0.27–0.79)**	115	11.3	**0.51 (0.26–0.97)**	79	0		37	0.0	
Travel												
Never/seldom	461	13.2	1.00	259	20.5	1.00	255	6.3	1.00	205	7.8	1.00
Often	376	11.2	0.78 (0.56–1.09)	208	17.3	0.79 (0.55–1.13)	200	7.5	1.19 (0.57–2.52)	159	9.4	1.10 (0.62–1.97)
Ever married												-
Single	700	9.6	1.00	331	15.7	1.00	187	3.7	1.00	98	7.1	1.00
Married	139	27.3	**2.39 (1.40–4.05)**	137	27.7	**1.82 (1.07–3.10)**	268	9	1.39 (0.49–3.95)	266	9	0.91 (0.34–2.47)
Ever had sex												
No	370	4.1	1.00	-	-	-	90	0	**a**	-	-	
Yes	468	19.2	**4.52 (2.42–8.42)**				364	8.5				
Ever given birth												
No	648	9.1	1.00	279	15.8	1.00	186	1.1	1.00	97	2.1	1.00
Yes	189	24.3	**2.25 (1.36–3.69)**	189	24.3	1.50 (0.91–2.48)	269	10.8	**8.78 (1.86–41.3)**	267	10..9	**4.90 (1.07–22.4)**
Number of lifetime sexual partners^b^												
0 partner^5^	370	4	1.00	-			90^b^	0	-			
1 partner	247	17.8	**4.43 (2.31–8.53)**	247	17.8	1.00	186	6.5	0.43 (0.17–1.07)	186	6.5	1.00
2 partners	125	20	**4.86 (2.31–10.2)**	125	20	1.06 (0.61–1.86)	108	9.3	0.63 (0.24–1.66)	108	9..3	1.37 (0.56–3.31)
≥ 3 partners	88	23.9	**6.13 (2.82–13.3)**	88	23.9	1.34 (0.73–2.43)	65	13.8	1.00	65	13..8	2.01 (0.79–5.12)

(i) Categorization:

i. Cluster educational levels: urban – low (9.0–10.5 years), middle (10.6–11.0 years), high (11.1–11.3 years); rural – low (4.0–5.3 years), middle (5.4–6.5 years), high (6.6–9.5 years)

ii. Cluster employment: urban – high (30–36%), middle (37–40%), low (41–46%); rural – high (36–46%), middle (47.61%), low (62–77%)

iii. Individual level education: urban – low (grade 0 – 7), middle (grade 8 – 11), high (grade 12 and above); rural – low (grade 0 – 4), middle (grade 5 – 7) & high (grade 8 and above)

^(2) ^AOR = age-adjusted odd ratios ^(3) ^Significant results are in bold (p < 0.05) ^(5) ^'0 partner' is equal to the number young women who have not had sexual intercourse

^a ^AOR could not be computed because the prevalence for one of the elements was zero

^b ^For all women in the rural area, we used "≥ 3 partners" as the reference since the prevalence among those with "0 partners" was zero

**Table 2:** Multivariate analysis of HIV infection among young urban women aged 15–24 years

Variables	All women		Sexually active women only	
	
	Underlying factors	With proximate factors	Underlying factors	With proximate factors
**Cluster-level variables**				
				
Education^1^				
Low	1.00	1.00	1.00	1.00
Middle	0.85 (0.50–1.44)	0.93 (0.55–1.60)	1.08 (0.60–1.95)	1.08 (0.60–1.94)
High	0.57 (0.32–1.02)	0.60 (0.33–1.07)	0.64 (0.33–1.23)	0.63 (0.33–1.21)
**Individual-level variables**				
Education^2^				
Low	1.00	1.00	1.00	1.00
Middle	0.73 (0.41–1.28)	0.70 (0.40–1.24)	0.80 (0.43–1.49)	0.79 (0.42–1.49)
High	**0.35 (0.19–0.62)**	**0.37 (0.21–0.66)**	**0.43 (0.23–0.80)**	**0.44 (0.23–0.83)**
				
Current student				
Not student	1.00	1.00	1.00	1.00
Student	0.66 (0.37–1.17)	0.80 (0.45–1.43)	0.74 (0.37–1.45)	0.76 (0.38–1.50)
				
Ever married				
Single	1.00	1.00	1.00	1.00
Married	1.54 (0.89–2.66)	1.31 (0.76–2.27)	1.31 (0.75–2.28)	1.27 (0.69–2.33)
				
Ever had sex				
No		1.00		
Yes		**3.60 (1.87–6.93)**		
				
Ever given birth				
No				1.00
Yes				1.04 (0.58–1.85)
				
Number of lifetime sexual partners				
0 partner^5^				-
1 partner				1.00
2 partners				1.02 (0.57–1.82)
≥ 3 partners				1.11 (0.60–2.08)

^(1) ^Cluster level education is based on the mean years of educational attainment of the population in the neighbourhoods: – urban: low (9.0–10.5), middle (10.6–11.0), high (11.1–11.3)

^(2) ^Individual level education categorization is as follows: urban: low (grade 0 – 7), middle (grade 8 – 11), high (grade 12 and above)

^(3) ^CI, confidence interval

^(4) ^AOR, age-adjusted odds ratio

^(5) ^'0 partner' is equal to the number young women who have not had sexual intercourse

Significant results are in bold (**p < 0.05**)

**Table 3:** Multivariate analysis of HIV infection in young rural women aged 15–24 years

Variables	All women		Sexually active women only	
	
	Underlying factors	With proximate factors	Underlying factors	With proximate factors
**Cluster-level variables**				
				
				
Education^1^				
Low	1.00	1.00	1.00	1.00
Middle	0.49 (0.20–1.21)	0.49 (0.20–1.21)	0.44 (0.18–1.11)	0.41 (0.16–1,06)
High	**0.29 (0.09–0.87)**	**0.24 (0.09–0.87)**	**0.29 (0.09–0.90)**	**0.30 (0.10–0.93)**
				
**Individual-level variables**				
Education^2^				
Low	1.00	1.00	1.00	1.00
Middle	1.01 (0.42–2.40)	1.01 (0.42–2.40)	1.00(0.41–2.39)	0.97 (0.40–2.36)
High	1.37 (0.43–4.36)	1.37 (0.43–4,36)	1.25 (0.39–4.06)	1.35 (0.41–4.40)
				
Current student				
Not student				
Student	**a**	**a**	**a**	**a**
				
Ever married				
Single	1.00	1.00	1.00	1.00
Married	1.09 (0.36–3.35)	1.09 (0.36–3,35)	0.68 (0.23–2.06)	0.40 (0.13–1.26)
				
Ever had sex				
No			-	-
Yes		**a**		
				
Ever given birth				
No				1.00
Yes				**6.37 (1.24–32.7)**
				
Number of lifetime sexual partners				
0 partner^5^				-
1 partner				1.00
2 partners				1.05 (0.42–2.63)
≥ 3 partners				1.76 (0.67–4.61)

^(1) ^Cluster level education is based on the mean years of educational attainment of the population in the neighbourhoods: rural: low (4.0–5.3), middle (5.4–6.5), high (6.6–9.5)

^(2) ^Individual level education categorization is as follows, rural: low (grade 0 – 4), middle (grade 5 – 7) & high (grade 8 and above).

^(3) ^CI, confidence interval

^(4) ^AOR, age-adjusted odds ratio

^(5) ^'0 partner' is equal to the number young women who have not had sexual intercourse

^a ^AOR could not be computed because the prevalence for one of the elements was zero.

Significant results are in bold (**p < 0.05**)

To assess the pathways through which individual risk factors were likely to mediate the effect of neighbourhood educational level on HIV infection, we examined the association of neighbourhood educational level with individual variables using linear-by-linear association tests (also known as Mantel-Haenszel statistical test). However, this test does not adjust for the effect of data clustering, and thus the actual strength of associations may be weaker than estimated by the test. The individual factors were compared among the three levels (i.e., high, middle and low) of neighbourhood educational attainment in both urban clusters and rural clusters (Table 4). Median age at first sexual intercourse was estimated using the survival analysis command stsum. Log-rank test for equality of survivor functions was used to compare the median ages between groups. Median age and median lifetime sexual partners were compared via K-sample equality of medians test.

**Table 4:** Association of cluster educational attainment with other risk factors in young women

	Cluster educational attainment							
	
	Urban				Rural			
	
	Low	Middle	High	P value	Low	Middle	High	P value
All young women (n):	397	195	248		159	136	161	
Median age (yrs)	19	19	19	0.550^c^	20	20	19	0.886^c^
Current student (%)	36.7	35.1	48.0	**0.006**^a^	4.5	11.3	35.4	**0.000**^a^
Travelled often (%)	41.0	49.0	48.0	0.062^a^	38.0	41.2	52.2	**0.011**^a^
Ever married (%)	47.3	23.2	29.5	**0.000**^a^	34.9	29.8	35.3	**0.000**^a^
Ever sexually active (%)	61.9	49.5	51.2	**0.004**^a^	82.9	92.6	67.1	**0.000**^a^
Median age at 1^st ^sex (yrs)	18	20	20	**< 0.001**^b^	15	16	17	**< 0.001**^b^
Ever sexually active (n):	245	96	127		131	125	108	
Ever given birth (%)	45.5	36.8	35.2	**0.045**^a^	79.4	76.0	63.0	**0.005**^a^
Sex before age 16 (%)	35.8	31.6	24.0	**0.022**^a^	73.8	71.8	57.0	**0.007**^a^
Median lifetime sexual partner (n)	1.0	1.0	1.0	0.305^c^	2.0	1.0	2.0	0.147^c^
STI (%)	3.9	3.1	1.6	0.240^a^	9.1	3.6	2.9	**0.036**^a^

(a) Linear-by-linear association test

(b) K-sample equality of medians test

(c) Log-rank test for equality of survivor function

Significant results are in bold (**p < 0.05**)

The multivariate analyses were conducted by the use of a two-level mixed effects logistic regression model. Separate multivariate models were constructed for urban and for rural neighbourhoods. In addition, separate models were built for all women and for sexually active women only. For all women, underlying variables were entered first and then the 'ever had sex' variable was added. For the sexually active women, we added the factor 'ever given birth' and 'number of lifetime sexual partners'.

### Ethics

Ethical clearance for the population based-survey protocol was obtained from the University of Zambia Research Ethics Committee. Participation in the survey was based on informed written consent. Respondents were informed that the HIV testing based on saliva was for research purposes only and it was going to be handled anonymously. Those respondents that wanted to know their HIV status were offered voluntary counselling and testing (VCT) at home.

## Results

The HIV prevalence was 12.5% in the urban area and 6.8% in the rural area. In both urban and rural areas, the neighbourhoods with a low average educational attainment had higher HIV prevalence than neighbourhoods with a high average educational level, 15.9% vs. 10.7% and 7.3% vs. 3.7% respectively. The logistic regression analysis for cluster-level variables adjusted for age showed neighbourhood educational attainment to be negatively associated with HIV infection in both the urban and rural populations. The other neighbourhood variables, wealth index and employment, were not significantly associated with HIV prevalence, but the direction of the relationships were the same, i.e. higher socioeconomic status being associated with lower HIV prevalence (Table 1). Neighbourhood educational attainment was correlated with neighbourhood wealth (Pearson correlation coefficient 0.8 in the rural area and 0.45 in the urban area), with the presence of electricity in the households (0.6 in the rural and 0.14 in the urban area), and with neighbourhood employment rate (0.7 in the rural and 0.15 in the urban area).

Table 4 shows that in the urban area, women who resided in neighbourhoods with a low mean educational-level were more likely to engage in sexual activity early and not to be in school than those who resided in neighbourhoods with a high mean educational-level. Among those who were sexually active, women in neighbourhoods with low mean educational-level were more likely to engage in sex before age 16, marry early and give birth early compared to those in the neighbourhoods with high educational-level. A similar pattern was observed in the rural neighbourhoods. Generally, women in the rural area engaged in sexual intercourse earlier than their peers in the urban area, irrespective of the level of education in the neighbourhoods they resided. In terms of median lifetime partners, women in the rural clusters reported a higher number than the urban women, but there was no difference by educational level of the neighbourhood. The percentage reporting STI symptoms was highest in neighbourhoods with low educational level in the rural area, especially among young women aged 20–24 years.

The correlation between individual and cluster educational attainment was 0.17 in the urban and 0.53 in the rural area. In both urban and rural neighbourhoods, there was no evidence of an interaction between neighbourhood-level education and individual-level education. After adjusting for underlying individual factors in the multivariate analysis, the likelihood of young urban women in the neighbourhoods with a high average educational attainment to be infected with HIV was still lower than in less educated neighbourhoods, but the association was no longer significant (OR 0.57, 95% CI 0.32–1.02). Young women with higher education had a lower risk of HIV infection after controlling for the neighbourhood effect. Among sexually active urban women, neighbourhood educational level was not significantly associated with HIV infection in the multivariate analyses, whereas high individual-level education maintained its protective effect (Table 2).

In the rural clusters the significant association between neighbourhood educational attainment and HIV prevalence persisted even after adjustments for individual-level underlying and proximate variables in the multivariate analysis. When only sexually active young women were taken into consideration, the association between neighbourhood educational level and HIV remained closely the same (Table 3). In contrast to the urban neighbourhoods, individual education was not significantly associated with HIV prevalence among young rural women, but it appeared to be somewhat protective in the bivariate analysis (Table 1) when in fact it tended to be a risk factor in the multivariate analysis (Table 3).

## Discussion

We have previously reported a remarkable shift over time in the association between individual-level educational attainment and HIV infection among young people in Zambia [19,22], and similar observations have been revealed from other high HIV prevalence populations in Africa [28]. Educational attainment is a key marker of socio-economic position, and here we examined structural effects on HIV prevalence measured by the association between small area-level educational attainment and HIV prevalence independent of the respective individual-level of education. The magnitude of these effects was highest in the rural populations, i.e. OR 0.24, 95% CI 0.09–0.87 after adjusting for age and underlying individual variables. The respective urban OR was 0.57, 95% CI 0.32–1.02. These findings suggest structural effects on HIV prevalence.

There are many possible indicators of socio-economic position, both at the individual-level and at the neighbourhood-level. Frequently used indicators at the individual-level are occupation, income, educational attainment and wealth. These are seen as dimensions of socio-economic position. They are most often handled separately, but sometimes used as a basis for a construct measure [29]. Neighbourhood educational attainment seems to be a good proxy of the socio-economic position of the local areas due to the close correlation between this variable and average wealth index in the neighbourhoods. The associations between neighbourhood educational attainment and HIV infection and neighbourhood wealth index and HIV infection were similar in direction, and for the rural stratum, also compatible in strength. The strong correlation between neighbourhood educational attainment and the presence of electricity also suggests that the aggregate variable for educational attainment captures structural or contextual aspects of the neighbourhoods, although we cannot rule out that it reflects compositional differences between the neighbourhoods as well, i.e. that the differences between the neighbourhoods are produced by the kind of people who live there (a selection effect). The multivariate analyses showed that adjustment for individual underlying and sexual behaviour factors weakened the association between the educational level of the urban neighbourhoods and HIV infection. This indicates that the effect of neighbourhood educational attainment on HIV was partially mediated through these individual factors as conceptualised in the proximate determinants framework. In the rural area the association between HIV prevalence and average education in the neighbourhoods was not reduced after adjustment for individual factors. This may reflect that the most important mediating factors were not included in our model and/or that those included were measured with low validity. The finding that neighbourhood and individual educational attainment in the rural area tended to have contrasting associations with HIV prevalence (the first being protective and the second tending to be a risk factor) supports the assumption that the aggregate variable for neighbourhood educational attainment captures not only differences in education itself but also important differences in the socio-economic context of the neighbourhoods that may protect educated and wealthier neighbourhoods from HIV infection.

To what extent education in itself creates the differences in HIV prevalence between neighbourhoods is difficult to judge. Although neighbourhood educational attainment seems to be a good proxy of structural neighbourhood differences in the present data, we cannot rule out the possibility that education per se plays a role too. For example, it is believed that the spread of education in the community environment moulds socially acceptable behaviour [24]. Young women in the most educated neighbourhoods in this study seemed to delay sexual debut and marriage compared to those in the least educated neighbourhoods, and this may reflect differences in what is perceived to be socially acceptable. It could also reflect that women from educated neighbourhoods have a more independent position and are actively encouraged to pursue an education before establishing a relationship to a man and having children. Other studies suggest that in neighbourhoods with low average educational attainment or financially deprived areas adult supervision may be weaker [30]. As parental involvement may function as a social control and socialization mechanism in the family [30], limited parental involvement seems to be generally associated with a higher level of pre-marital sexual activity and problem behaviour [31]. This may be translated into higher chances of HIV infection among young women in the least educated neighbourhoods when compared with their peers who reside in areas with high average education. Further, educated women are more likely to marry educated men, and as educated men are less likely to engage in risky sexual behaviour [25] and they are less likely to be HIV infected, this probably also has an impact on the HIV risk of educated women.

Past HIV prevention efforts have been overly focused on individual behaviours, but the recognition of the need to combine behavioural, structural and biomedical approaches seems to be growing [32,33]. Although the observed shift in the association between educational attainment and HIV prevalence in several high prevalence African countries might be an example of substantial effects of preventive programmes focused on individual behaviour [19,22,25,34], these preventive programmes seem to have had limited impact on the less educated and impoverished part of the population. There is still limited understanding of why the less educated are not adhering to behaviour change messages. The most educated may respond faster to protect themselves due to higher concern for their health and stronger belief in their own self-efficacy as predicted by the framework "diffusion of innovations" [25,35,36]. Or it might be rooted in the pedagogical approach employed by the campaigns. Any elements of 'blame the victim' approaches to foster behaviour change messages may have been counter-productive or perceived as irrelevant by less educated persons [34]. The neighbourhood effects on HIV prevalence might indicate the need for combining programmes focussing on individual behaviour with structural approaches to address those factors that shape or constrain individual behaviour. An example of an important programme that may contribute to this is the Programme for the advancement of girls' education (PAGE), adopted in Zambia in the early 1990s, with the purpose to improve girls' access to school [37]. It seems that this programme has started to bear fruits in terms of substantial improvement in girls' school attendance at both primary and secondary level in the period 1990–2000 [38]. However, the net junior and senior secondary school attendance rate is still low in Zambia, particularly in rural (8% and 5% vs. 35% and 29% in urban areas) and low cost areas (31% and 23% vs. 50% and 49% in high cost areas)[39]. Increasing school attendance in low cost (poor) areas is likely to have an effect on HIV incidence as it may increase the self-efficacy of young people to protect themselves against HIV infection and increase their chances of obtaining formal employment and thus improve their own living conditions. Making secondary schools more available in rural areas may be one way to increase net school attendance, and removing school fees is another.

A number of factors may have contributed to biasing the neighbourhood effects in our study. In the first place, census clusters may not be adequate proxies of an individual's neighbourhood surroundings and thus may not adequately capture differences across neighbourhoods [8]. However, these areas are small enough to be useful proxies of the natural neighbourhoods. Furthermore, the number of clusters used by the study was low; i.e. 10 in each of the strata. This resulted in low power to detect in-between cluster variation in HIV prevalence[40]. There was also limited information on more specific neighbourhood characteristics found to be related to HIV prevalence in other studies, for example, availability of water, markets and health facilities [13] and availability of bars and proximity to the nearest big town [14]. Non-response may have affected our results if young women who were absent during the household visits had a different risk profile than those found at home (e.g. young women attending boarding school could engage in both less or more risky sex than their counterparts at home). However, the proportion absent (which amounted to 11–14% among women overall) was rather low and thus is not likely to have caused a major bias. The modest effect of adjusting for proximate variables may be due to bias in the self-reporting of sexual behaviour as women have a tendency to underreport stigmatized sexual behaviour experiences and over-report normative behaviour [41,42]. A particular example in this regard was the revealed 4.1% HIV prevalence among young urban women who reported never engaging in sexual intercourse. Since the epidemic in Zambia is mainly transmitted through heterosexual intercourse and the likelihood that women aged 15–24 in the pre-ARV era have survived with a HIV infection transmitted from the mother during pregnancy/infancy, is extremely low, this indicates a certain underreporting of sexual activity. Other studies have also discovered current pregnancy, HIV and sexually transmitted infections among young people who denied having sex [2,42,43]. Some of the young women who denied having had sex may have been raped or forced to have sex and denied sexual activity, as they had not participated voluntarily. A study in Kenya found that half the adolescent girls who claimed that they had never had sex, reported having been forced to have sex [44]. Ten percent of Zambian women aged 15–19 had been forced to have sex against their own will according to the Zambia Sexual Behaviour Survey 2005 [45]. Finally, a cross-sectional survey does not allow determination of whether exposure to neighbourhood factors precedes the development of the outcome.

## Conclusion

In conclusion, the findings suggested neighbourhood contextual effects on HIV prevalence in these very HIV prevalence areas. Neighbourhood educational attainment appeared as a good proxy of the socioeconomic position and is likely to capture a complexity of overlapping contextual factors. In this regard the strength of the local educational system seems to be a case in point, supporting the value of ongoing efforts to improve access to secondary education particularly for women. However, more complex structural approaches will be needed to reduce HIV risk and vulnerability. Future research should include more neighbourhood factors of relevance to HIV prevalence as part of the effort to better understand the causal mechanisms involved.

## Competing interests

The authors declare that they have no competing interests.

## Authors' contributions

NK participated in the analysis and interpretation of the data and led the drafting of the manuscript. IFS actively participated in interpreting the results as well as revising the manuscript. KF conceptualised and designed the survey, co-ordinated and supervised the data collection, and took an active part in the analysis, interpretation of the results and revising the manuscript. All authors read and approved the final manuscript.

## Pre-publication history

The pre-publication history for this paper can be accessed here:


